# Modification patents across multiple NDAs in pharmaceutical protections

**DOI:** 10.1093/haschl/qxag123

**Published:** 2026-05-21

**Authors:** Robin Feldman, Ramy Alsaffar, Tanziuzzaman Sakib

**Affiliations:** Center for Innovation (C4i), University of California Law, SanFrancisco, San Francisco, CA 94102, USA; Center for Innovation (C4i), University of California Law, SanFrancisco, San Francisco, CA 94102, USA; Center for Innovation (C4i), University of California Law, SanFrancisco, San Francisco, CA 94102, USA

**Keywords:** drugs, generic, drug industry, patents as topic, legislation, drug, drug approval process, new drug approval process, economics, pharmaceutical, health policy

## Abstract

**Introduction:**

Our study examines modification patents across multiple New Drug Applications (NDAs), employed by pharmaceutical companies to extend patent protections. Ours is the only comprehensive study of modification patents by Active Pharmaceutical Ingredient (API) across multiple NDAs, the approach of current regulatory initiatives, investigating how these patents extend drug protections and impede generic entry.

**Methods:**

We examine 1028 modification patents, categorizing them as chemical variations, formulation changes, treatment methods, or device/agent. We tabulated additional granted protection time, correlations with new NDAs, and litigation from potential generics.

**Results:**

On average, 5 modification patents protected each drug, extending granted protection periods by 10.9 years. Examining market outcomes for the 199 drugs with generics approved by August 2025, or whose primary patents had expired by then, modification patents provided a median of 2.3 years' median effective additional protection time before generic entry. Eighty-nine percent of drugs' protection periods were extended by modification patenting in some dimension. Fifty-eight percent had a modification patent associated with a new NDA, and 70% had at least 1 modification patent in Paragraph IV-related litigation.

**Conclusion:**

These findings suggest we reconsider societal resources dedicated to modification patenting, generally and during clinical decision-making. Hope for regulatory and legislative reform lies in a “one-and-done” policy and restricting certain patent types.

## Teaser

When investigating modification patents, scholars have tracked drugs at the level of New Drug Applications (NDAs) or by examining only a few Active Pharmaceutical Ingredients (APIs). Modern legislative and regulatory efforts examine drugs by API to better capture strategic behaviors. Therefore, obtaining data by API is imperative to mirror regulatory institutions and legislative efforts. Our study is the most comprehensive, up-to-date examination of modifications at the API level across multiple NDAs. We hypothesized that each additional patent would extend a drug's exclusivity period. Additional patents erect barriers to competition simply by listing in the Orange Book, and listing ensures a competitor can be subject to an automatic 30-month application stay if the brand manufacturer decides to sue. This delay creates friction that may maintain high prices for consumers in the absence of lower-priced competition. We also investigated how these patents played out after listing: We examined the frequency of new NDAs resulting from modification patents; whether modification patents erected an additional barrier to entry, as measured by approval delays and litigation costs; and, if generics were able to enter the market, how quickly.

Key points

- Ours is the first comprehensive study to align with current regulatory and legislative initiatives by tracking all patent protections that apply to an API, across multiple NDAs.
- On average, each drug was protected by 5 modification patents, extending the granted protection period by 10.9 years beyond the primary patent's expiration date. For the 199 drugs with generics approved by August 2025, or whose primary patents had expired by that time, modification patents provided a median of 2.3 years of effective additional protection time. Overall, 89% of the drugs' right to exclude was extended by modification patenting in some dimension.
- Fifty-eight percent of the drugs had a modification patent leading to a new NDA, and 70% had at least 1 modification patent involved in Paragraph IV-related litigation.

## Introduction

This study examines the prevalence and effects of modification patents. Our definition of modification patents largely overlaps with what scholars have variously described as secondary patenting, tertiary patenting, and evergreening—which all involve modifying medications without changing their Active Pharmaceutical Ingredient (API(s)). Yet, the uses of these terms and their definitions are myriad.^1^ Instead, we specifically define modification patents by using the following categories, generally adhering to the groupings developed by Kapczynski,^2^ the UN,^3^ and the European Commission:^4^ (1) chemical variation patents, (2) formulation change patents, (3) method of treatment patents, and (4) device or agent patents. We note that unlike Kapczynski, we include a category of device or agent patents.

Modification patents' role in pharmaceutical innovation is contested by some scholars who believe such patents provide limited innovative contributions while playing a role in the cost of prescription medication.^5,6^ Consider, for example, a formulation-change patent on an in-active ingredient, such as an adjustment to a pill's taste. Although such changes may not alter the API, they can affect whether a generic version of the drug is considered therapeutically substitutable. Because generic entry often depends on automatic substitution, differences introduced by these patents would block the generic company from making a substitutable version. Thus, modification patents can shape the market's competitive landscape by broadening brand companies' rights to exclude and hindering generics' pathways to providing lower-priced alternatives. We note that some scholars have argued through case studies that the innovative value of secondary patenting is underestimated.^7^ We do not comment on specific cases, some of which may be deserving of protection, but we note that such studies do not negate evidence of a broader pattern.

When investigating modification patents, scholars have tracked drugs at the level of NDAs (New Drug Applications)^8^ or by examining a few APIs.^9,10^ NDA level analysis treats each approved application as a discrete drug, with the result that new versions of a drug issued under subsequent NDAs are not identified as part of a single drug's patent portfolio. In contrast, API level analysis groups all NDAs sharing the same active ingredient, yielding a comprehensive view of the cumulative patent protection issued to a drug throughout its lifecycle. This distinction is particularly important for identifying product hopping, a management strategy whereby manufacturers shift patients to a modified version of a drug, often approved under a new NDA, shortly before generic entry, thereby extending market exclusivity while obscuring the continuity between products. Tracking drugs at the NDA level risks treating these successive versions as unrelated products, concealing the full scope of such strategies.

Recent regulatory approaches likewise aggregate across NDAs. The 2023 Inflation Reduction Act^11^ and the Centers for Medicare and Medicaid Services (CMS)^12^ examine drugs by API to reveal how each drug is modified across different NDAs. Under the 2023 Inflation Reduction Act, CMS defines a “drug” to include all dosage forms and strengths with the same active ingredient and NDA holder, including products marketed under different NDAs.^11,12^ Commentary on the program has noted that this aggregation captures multiple formulations of the same drug and limits fragmentation across products.^13^ By adopting an API level framework across multiple NDAs, our study aligns with this broader analytical approach and provides the most comprehensive, up-to-date examination of modification patenting across a drug's lifecycle.

We hypothesized that each additional patent would extend a drug's exclusivity period. Additional patents erect barriers to competition simply by listing in the Orange Book, and listing ensures a competitor can be subject to an automatic 30-month application stay if the brand manufacturer decides to sue.^14^ This delay creates friction that may maintain high prices for consumers in the absence of lower-priced competition.^15^ We also investigated how these patents played out after listing: We examined the frequency of new NDAs resulting from modification patents; whether modification patents erected an additional barrier to entry, as measured by approval delays and litigation costs; and, if generics were able to enter the market, how quickly.

These dynamics have implications deeper than system-level market analysis. Because generic uptake relies in part on prescribing practices and eligibility for automatic substitution, the types of patents examined in this study ultimately can influence which products are dispensed to patients. As a result, physicians play a role in how these dynamics translate to patient access and costs, so doctors have the potential to affect their proliferation. We hope this study provides doctors with more information to better navigate the drug landscape and care for patients, and policymakers with more insights into potential systemic reforms.

## Data and methods

### Data sources and study scope

This analysis relied on data obtained from the following databases: The Orange Book (October 2019 version),^16^ the Derwent Innovation patent search platform,^17^ Drugs@FDA Database,^18^ Patent Litigation Docket Reports Data (“USPTO”),^19^ NCBI's PubChem database,^20^ BloombergLaw Docket Search,^21^ the US Food and Drug Administration's (“FDA”) list of New Drug Applications (“NDAs”) since reclassified as Biologics License Applications (“BLAs”),^22^ and the Evergreen Database containing information regarding Orange Book-listed patents between 2005 and 2015.^23^

As discussed above, this study defines a drug by its API(s), consistent with regulatory definitions. When the FDA has approved multiple NDAs for a given API(s), we consider each NDA to be a different version of the same drug.

The study draws on a foundational dataset constructed to identify instances in which modification patenting accompanied the issuance of new NDAs for the same API. That dataset was assembled by identifying APIs associated with multiple NDAs in the Orange Book and manually reviewing the independent claims of all patents listed for those APIs using the Derwent Innovation patent search platform. Each patent was categorized as either a primary patent (covering the original chemical compound) or a modification patent.

Because this manual claim review and classification process defines the structure of the foundational dataset, it also determines the scope of drugs included in the analysis. In particular, the dataset includes APIs associated with multiple NDAs and does not include APIs with only a single NDA, which were not subjected to manual patent claim review as part of the original study design. Within the Evergreen Database containing drugs listed in the Orange Book between 2005 and 2015, approximately 30% of APIs are associated with multiple NDAs, while the remaining APIs have only a single NDA. The present study therefore examines a defined subset of drugs for which detailed patent-level classification is available.

Our study was limited to drugs that: (1) are small molecule;^24^ (2) had multiple NDAs approved for their API(s); (3) had at least 1 product labeled as Rx in the Orange Book; and (4) did not have discontinued NDA(s). All APIs that met our inclusion criteria also had at least 1 modification patent listed in the Orange Book during the study period; no APIs were excluded on this basis. When calculating the effect of modification patents on generic competition, we eliminated the 4 drugs for which the primary patent had not expired as of September 2025, and that did not have generics, in order to eliminate cases when we could not ascertain whether it was the primary patent or the modification patent serving as a barrier to generics. In addition, we further segmented the drugs into: (1) drugs that had their earliest approved generics before the expiration of the primary patent; (2) drugs that had their earliest approved generics after the expiration of all primary patents but before the expiration of all modification patents; (3) drugs that had their earliest approved generics after the expiration of all patents; and (4) drugs that had no generics approved by August 2025 and whose primary patents had expired.

We used the Evergreen Database to catalogue all patents that were listed in the Orange Book between 2005 and 2015 for the included drugs. We analyzed each of those patents, excluding reissue patents, patents with reexamination certificates, and likely continuation patents—that is, patents that shared the same priority date, title, and expiry date.

In interpreting patent timelines within this dataset, it is important to note that patent terms may be extended or adjusted through mechanisms such as patent-term extensions (compensating for regulatory delay) and patent-term adjustments (compensating for administrative delay). As a result, patents associated with a given drug do not necessarily expire in the order in which they were filed, and modification patents may in some cases expire before the corresponding primary patent. This structural feature of the patent system is reflected in the dataset analyzed here.

Modification patents expiring prior to the primary patents may reflect the complexities of assembling a patent portfolio, including the difficulty of predicting the timing of USPTO processes, the potential for patent extensions, patent litigation that can lead to a judgement of invalidity or noninfringement of the patent or a settlement in which the generic enters prior to the expiration of the patent, and the potential advantages of multiple patents covering the same period of time.

Finally, we used USPTO's Patent Litigation Docket Reports Data and complaint files from the BloombergLaw Docket Search platform to identify patents subject to Paragraph IV certifications and consequently involved in district court litigation for infringement and invalidity (including declaratory judgment).

### Categorizing patents

As discussed above, we largely followed the patent groupings used by the UN, the European Union, and adopted by Kapczynski.

**Figure 1:**
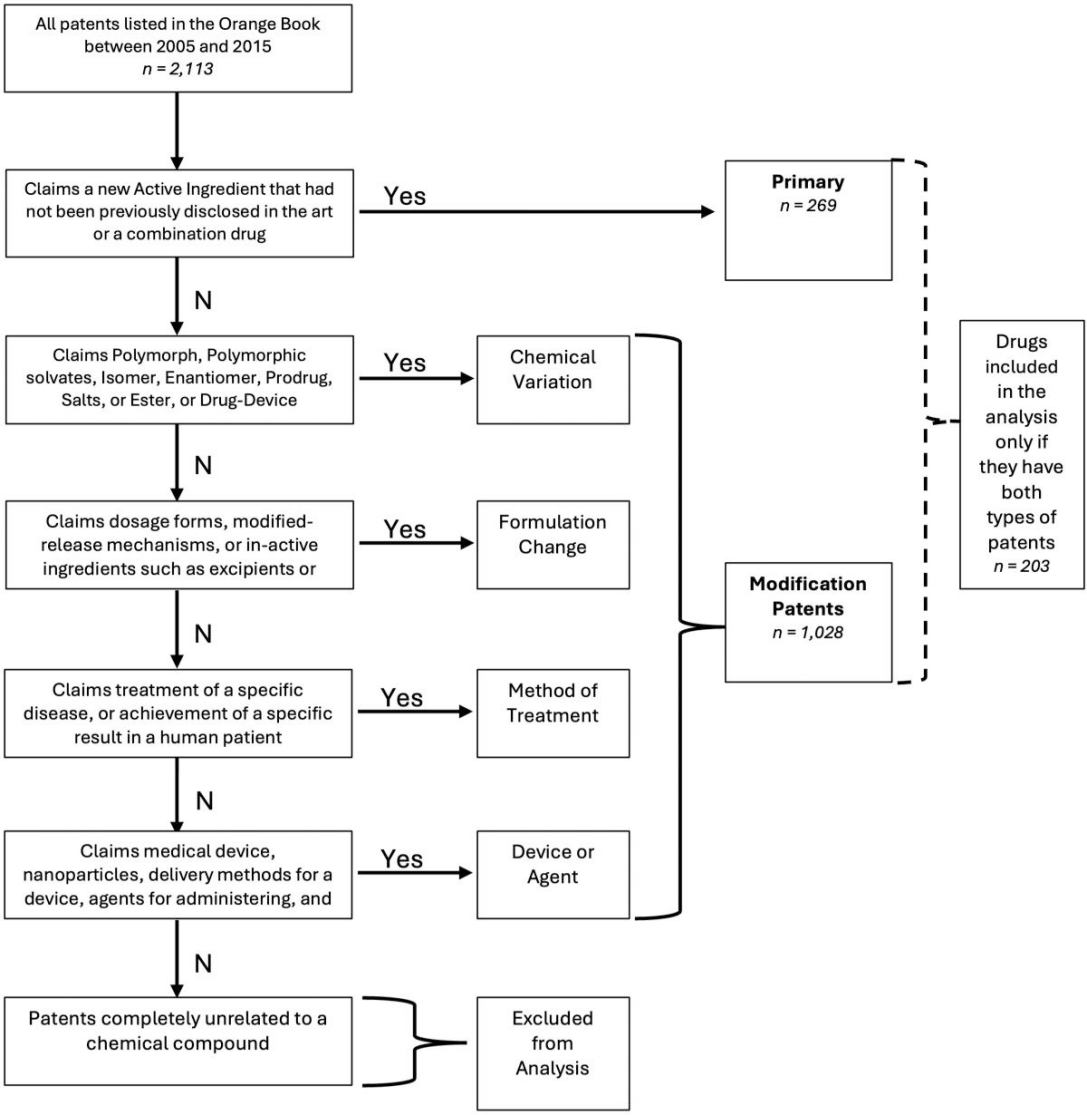
Hierarchical categorization of patents analyzed in this study. We restricted our study to drugs with at least 1 primary patent and 1 modification patent. Our analysis relied on the earliest- and latest-expiring primary patents for each drug. The vast majority of drugs (96.6%) had a single primary patent, while a small minority (3.4%) had 2; no drug had more than 2 primary patents. In cases with multiple primary patents, we used the latest expiration date as the baseline, yielding a conservative estimate of additional protection attributable to modification patents.

We then manually reviewed the independent claims of every patent included in our study and categorized them into: (1) primary patents; and (2) modification patents. Building on this classification, we further categorized modification patents into the following subcategories: (i) minor chemical variation, (ii) formulation change, (iii) method of treatment, and (iv) device or agent (See Figure 1).

Patent classification was determined by the highest-ranked independent claim identified through a hierarchical decision framework based on patents, before reconsolidation into drugs. This structure avoided concerns of patents containing multiple types of claims (eg, both chemical variation and formulation change) being classified only once, thereby obscuring the presence of lower-ranked claim types within the same patent. This avoidance was possible because the classification procedure operates at the patent level, whereas the outcome consolidates patents at the drug level—as a result, regardless of how an individual patent is categorized within the hierarchy, the total number of drugs included in the analysis remains unchanged.

## Results

### Prevalence of modification patents

Our analysis found that an average of 5 modification patents protected each of the 203 drugs in our study. On average, only 1 of those 5 patents expired before the expiration of the drug's primary patent(s) while 4 expired after that expiration date. Moreover, 166 of the drugs (81.1%) had more than 1 modification patent protecting them with 25 drugs (12.4%) listing 10 or more such patents.

Of the 1028 modification patents analyzed, 329 patents (32%) were formulation change patents, method of treatment patents accounted for 311 patents (30.3%), device or agent patents accounted for 294 patents (28.6%), and chemical variation patents accounted for 94 patents (9.1%) (see Table 1). This study's classification approach (ie, assigning each patent to a single highest-ranked category) may underestimate the prevalence of patents with multiple subcategories, including lower-ranked categories due to those patents' classification in higher-ranked ones. However, the empirical distribution of the subcategories does not reflect a strong bias, as there is no observed inflation of the high-ranked categories than the lower ones, nor is there a gradual tapering in frequency from highest- to lowest-ranked categories.

**Table 1:** Frequency of subcategories and claim types of modification patents.

Subcategory	Claim type	Count	Subcategory total	Subcategory percentage
Formulation Change	Dosage Forms	262	329	32%
	Delayed or Modified Release	25		
	In-Active Ingredients	42		
Method of Treatment	Method of Treatment	311	311	30.3%
Device or Agent	Medical Device and Nanoparticles	124	294	28.6%
	Agent for Administrating	169		
	Kit	1		
Chemical Variation	Polymorph or Polymorphic Solvates	44	94	9.1%
	Isomer or Enantiomer	12		
	Prodrug	0		
	Salts or Ester	13		
	Drug Device Combination	25		
Total			1028	100%

In addition to their prevalence, modification patents varied in how their expiration timelines related to the corresponding primary patents. On average, only 1 of those 5 patents expired before the expiration of the drug's primary patent(s), while 4 expired after that date. Across all modification patents analyzed, 255 of 1028 (24.8%) expired before or at the primary patent expiration date, consistent with the variation in patent term lengths described above.

### Impact of modification patents on period of granted protection

Of the 203 drugs we studied, only 22 (11%) had a primary patent as their last expiring patent. In other words, 9 out of every 10 drugs analyzed here extended their period of granted protection by obtaining modification patents. We define the period of granted protection as the time between the drug's earliest approval and the expiration of its last-expiring patent, reflecting the full duration of protection afforded by the patent system. To measure the additional granted protection associated with modification patents, we calculated the time between the expiration of each modification patent and the later of (1) the date the patent was added to the Orange Book or (2) the expiration of the corresponding primary patent. On average, the additional granted protection period was 10.9 years, with some drugs receiving as much as 30.7 additional years. We break down the average additional protection time by subcategory of modification patent in Table 2.

**Table 2:** Average additional granted protection period, as measured by patent expiration dates, for each subcategory of modification patents.

Subcategory	Expires after the primary patent (%)	Average additional protection period (years)	Average additional protection period added at conclusion of all patents (years)
Chemical Variation	81%	6.6	6.8
Formulation Change	90%	8.3	10.3
Method of Treatment	71%	8.0	8.8
Device or Agent	61%	6.6	7.8

Given that multiple modification patents may contribute to a drug's additional granted protection period, the average additional-protection period granted to a drug can be higher than the average additional-protection period added by each subcategory of modification patents. Subcategories are in no particular order, and the table does not imply a filing sequence. Effective exclusivity periods may be shorter than the granted exclusivity periods reported here.

### Generic competition

Because protection as defined by patent terms does not necessarily correspond to market entry timing, we also examine how these protections operate in practice by considering the timing of generic competition. In other words, we measure not only whether modification patents created a barrier but also the extent to which generic companies were able to overcome that barrier. Of the 203 total drugs in our study, 68 (33.5%) did not have an FDA-approved generic alternative as of September 2025. Four out of those 68 drugs had primary patents that had not expired by September 2025 and did not have an Abbreviated New Drug Application (ANDA); we excluded these drugs from our analysis, as we explained in the Methodology section. For the 199 total drugs in our study that had generics as of August 2025, or whose primary patents had expired as of August 2025, the median additional affective protection time afforded by modification patents was 2.3 years.

We further segmented the data to specify the median protection conferred by modification patents in the different possible situations of generic entry timing. For (1) drugs with earliest approved generics before the expiration of primary patents, the median effective additional protection time was −6.6 years. We note that this number has limited interpretive value, since it reflects a period within which generic competition was already present. For (2) drugs that had earliest approved generics after the expiration of primary patents but before the expiration of modification patents, the median effective additional protection time was 2 years. For (3), the 13 drugs that had their earliest approved generics after the expiration of all patents, the median effective additional protection time was 4 years. Lastly, for (4) drugs with no generics approved as of August 2025, and whose primary patents had expired, the median effective additional protection time was 13.1 years. These results are illustrated in Table 3.

**Table 3:** Median effective additional years of protection, as measured by the time between primary patent expiry and the first generic approval date, or in the case of (d), the time between primary patent expiry and August 1, 2025.

Segment according to time of generic entry	Drug count	Median effective additional protection time in years
Generic entered prior to primary patent expiry	53	−6.6
Generic entered during additional protection period	69	2
Generic entered after all patents had expired	13	4.1
No generic, primary patent has expired	64	13.1
Total	199	2.3

In total, we identified 1553 generics that were therapeutically equivalent to a drug in our study. Only 275 of those generics (17.7%) received FDA approval before the primary patents of the corresponding drug expired, while 910 generics (58.6%) received FDA approval after primary patent expiry, when only modification patents remained in force. The remaining 368 (23.7%) were approved after the expiration of all patents protecting the corresponding drug.

### Modification patents leading to new NDAs

We identified instances in which modification patents were associated with the issuance of subsequent NDAs for already-marketed drugs, a pattern that can be associated with product hopping. We did so by locating patents that claim modifications to a drug's patent and that (1) were not listed under a drug's first-approved NDA, and (2) were first added to the Orange Book for a subsequent NDA within 3 months of the day that NDA was approved. We found 118 drugs in this study (58%) had at least 1 modification patent linked to the issuance of a new NDA. These findings reflect patterns within drugs with multiple NDAs, the population examined in this study. Readers should note that a drug may have multiple subsequent NDAs and that multiple modification patents may be added to the Orange Book within 3 months of the day the new NDA was approved. The rationale behind this investigation and the results are further explored in the Discussion section.

### Paragraph IV certification and associated litigation

We measured the effect of Paragraph IV certifications and related litigations on the regulatory approval timeline for the ANDAs that are therapeutically equivalent to 1 of our 203 drugs. Of 1553 such ANDAs, 362 had their approval letters available on the Drugs@FDA database. One hundred and ninety-two of those ANDA approval letters contained at least 1 Paragraph IV certification and included the date when the ANDA was accepted for review by the FDA (See Table 4). In 60.4% of the cases (116 out of 192), the brand manufacturer initiated infringement litigation against the ANDA applicant, while in 37.5% of the cases (72 out of 192), the brand declined to sue. Litigation status was unclear in the remaining 4 cases.

**Table 4:** Frequency of subcategories and claim types of modification patents that were involved in Paragraph IV litigation.

Subcategory	Claim type	Count	Subcategory total	Percentage of patents that were involved in Paragraph IV litigation
Formulation Change	Dosage Forms	130	162	49.2%
	Delayed or Modified Release	12		
	In-Active Ingredients	20		
Method of Treatment	Method of Treatment	140	140	45%
Device or Agent	Medical Device and Nanoparticles	14	89	30.3%
	Agent for Administrating	74		
	Kit	1		
Chemical Variation	Polymorph or Polymorphic Solvates	27	46	48.9%
	Isomer or Enantiomer	6		
	Prodrug	0		
	Salts or Ester	7		
	Drug Device Combination	6		
Total			437	42.5%

In other words, these patents were subjects of Paragraph IV certifications and were consequently involved in district court litigations for invalidity or infringement (including declaratory judgements).

Furthermore, we found that when an ANDA contained Paragraph IV certifications, but the brand declined or failed to sue for infringement, the average time to FDA approval was 3.89 years with a median of 3.36 years. When the brand filed litigation against an ANDA with Paragraph IV certification, however, approval took substantially longer—5.23 years on average (median: 4.41 years). ANDAs facing Paragraph IV litigation thus took over a year longer to receive FDA approval than those that did not, with the approval-time distribution shifted later for the litigated group. We confirmed this using the Mann–Whitney *U* test, which compares whether 1 group's values tend to rank higher than the others, and the Kolmogorov–Smirnov test, which assesses whether 2 distributions differ in shape or location. Both yielded statistically significant differences (*P* < 0.01) (Mann–Whitney *U* = 5694, *n*_1_ = 116, *n*_2_ = 72, *P* < 0.01; Kolmogorov–Smirnov *D* = 0.296, *P* < 0.01).

Overall, median approval time for ANDAs with Paragraph IV certifications was 3.99 years, compared to 2.69 years for ANDAs whose approval letters did not mention such certification. Looking more broadly at ANDAs with Paragraph IV certifications, the median approval time was 3.99 years, compared to 2.69 years for ANDAs whose approval letters did not mention such certifications. In other words, ANDAs with Paragraph IV certifications were approved more than a year later than those without such certifications. This difference was statistically significant (Mann–Whitney *U* = 19836.5, *n*_1_ = 192, *n*_2_ = 155, *P* < 0.01; Kolmogorov–Smirnov *D* = 0.371, *P* < 0.01). Readers should note that the absence of any reference to Paragraph IV certifications in an approval letter does not necessarily imply that the ANDA submitted no such certifications.

Finally, we calculated how often the drugs and patents included in our study were involved in litigation arising from a Paragraph IV certification. We found that 142 of our 203 drugs (70%) had at least 1 modification patent that was the subject of a Paragraph IV certification and consequently involved in litigation. We found that litigation was common across the drugs in our sample: 142 of our 203 (70%) had at least 1 modification patent that was the subject of a Paragraph IV certification and consequently involved in litigation. By comparison, a smaller cohort of 102 drugs (50.2%) had at least 1 primary patent that was involved in litigation arising from Paragraph IV certifications. At the patent level, 42.5% of the modification patents (437 out of 1028) and 46.1% of the primary patents (124 out of 269) were involved in district court litigation arising from Paragraph IV certifications. Table 4 details the frequency of subcategories and claim types of modification patents that were involved in Paragraph IV litigation.

## Discussion

Our results present a window into the effects of modification patents across multiple NDAs. We found that each drug was protected by, on average, 5 modification patents, which together added an average of 10.9 years of additional granted protection beyond the primary patent's expiration. Furthermore, 9 out of 10 drugs had their period of granted protection extended beyond the expiry of their primary patent by a modification patent. These findings reflect the extent to which modification patents expand the duration of granted protection as defined by patent terms.

Our analysis of generic entry provides a complementary perspective on how these protections operate in practice. Across the 199 drugs in our study that either had a generic and/or expired primary patents by August 2025, the median additional effective protection time conferred by modification patents was 2.3 years. This finding highlights that the duration of granted protection does not fully align with market dynamics and that generics are able to overcome the barrier of granted protection, at least to some extent. Nonetheless, across both regulatory and market-based measures, modification patents extending beyond primary patent expiry are associated with additional periods of protection. Our key finding that modification patents added 2.3 years of additional effective protection time could have major consequences for both producers and consumers.

Although simply listing in the Orange Book is a deterrent to generics, we considered whether modification patents are litigated, given that litigation is widely understood to increase costs and may signal that listed patents are being contested as improperly granted or applied. We found widespread litigation: 42.5% of all modification patents in the study faced district court litigation arising from Paragraph IV certifications. With 70% of the drugs in the study having at least 1 modification patent involved in such litigation, these findings indicate that litigation—and its accompanying costs—is not confined to a narrow subset of drugs. In comparison, 50.2% of drugs have at least 1 primary patent involved in litigation arising from Paragraph IV certifications, indicating that the litigation burden associated with modification patents is comparable to, and may in some cases exceed, that associated with primary patents across the drugs in our sample.

Our findings are consistent with the well-documented practice of patent thicketing, in which pharmaceutical companies attain groups of patents to create overlapping protections around their drugs,^25^ thereby raising the cost, complexity, and risk of litigation for potential generics. These additional dimensions can stretch out the litigation timeline: we found that ANDAs involved in Paragraph IV litigation took 5.23 years to be approved, 1.34 years longer than those that were not.

Over half (58%) of the drugs we studied had a modification patent associated with the issuance of a new NDA. This may suggest evidence of product hopping, a technique employed by brand-name drug makers who shift consumers to a new NDA through targeted marketing. In particular, our finding that drugs with no unexpired primary patents, and no generics approved by August 2025, had a median protection time of 13.1 years, may indicate a pattern of successful product hopping, since no generics entered the market. Given that generic companies depend on pharmacy substitution for their sales flow, the product-hopping strategy ensures there will not be substitutable prescriptions written and allows the original company to avoid losing protection when their initial patents expire.^26^ The Inflation Reduction Act's Medicare Drug Price Negotiation program,^11^ as initiated by the Biden Administration and continued by the Trump Administration, takes this behavior into account by defining drugs for inclusion based on APIs, thereby grouping hopped products together. Thus, manufacturers cannot evade selection for negotiated prices through new formulations across NDAs.

Our study is not without limitations. We only looked at small-molecule drugs, and we considered only drugs that had more than 1 NDA. Thus, the study likely underestimates the results, given that life-cycle management strategies can occur within a single NDA. We also categorized the patents based on the highest-ranked independent claim within our classification scheme, which may underestimate the prevalence and impact of modification patents in other claim categories. However, the absence of a rank-ordered distribution indicates that the classification scheme does not meaningfully skew the observed proportions. In addition, our source for litigation data—the USPTO's Patent Litigation Docket Reports Data—only covers lawsuits filed on or before 2020, and BloombergLaw Docket Search failed to locate complaints for a few lawsuit files, likely underestimating the number of patents involved in Paragraph IV litigation. We also excluded reissue patents and patents with reexamination certificates, as the Derwent Innovation database did not include information on those patents, and device or agent patents that were unrelated to any chemical compound. When a patent was listed under multiple drugs, our analysis considered each case to be a separate patent, for ease of calculation. As a result, our list of 1028 patents includes 986 unique patents—that is, 42 patents (4%) were duplicate patents.

Fifty-three drugs in our study faced generic competition before their primary patents expired. This figure may include generics that entered prior to primary patent expiration due to Paragraph IV certifications or other forms of patent litigation, such as declaratory judgment. While it is beyond the scope of the study to attribute causality, we urge further research to determine the legal pathways responsible for this degree of generic entry prior to primary patent expiration.

We also note that scholars may differ on the value offered by different categories of modification patents. For example, method-of-treatment patents allow a drug to reach a new population, potentially providing value to the public, and the clinical studies needed to validate a drug's use in different populations are costly. Society could conclude, however, that expanding to new populations is sufficiently obvious to be unpatentable or would be best incentivized through a more limited reward than patenting.

### Possible solutions

The findings identified in this study have implications for how pharmaceutical markets function in practice and which medications are available and accessible to consumers. Although policy and regulatory approaches play a central role, other stakeholders also shape these dynamics. Medical professionals, for example, have multiple points of impact: as consumers who buy from pharmaceutical companies themselves or to stock in their offices; as prescribers to patients; as a lobbying force, and as partners with the pharmaceutical industry in advisory roles or through clinical evaluations. In each role, they should be aware of strategic behaviors that may restrict competition^27^ and help maintain prices.^28^ For example, a prescribing physician might ask: Is the most recent variant of the drug more effective for my patient? Can my patient afford it? If medical professionals pause to ask these questions before buying, prescribing, or encouraging the production and sale of drugs with modification patents, they could potentially slow the demand for such products, providing the opportunity to advance the interests of patients.

Considering the amount of societal time and resources expended for modification patents, including at a minimum at the patent office, in the courts, and by potential competitors, policymakers might consider whether the value justifies the cost, particularly when the extent of improvement may vary. In this context, some policymakers have proposed various versions of a “one-and-done” policy, in which a drug can have only a limited number of protections or a limited period of additional protections. For example, a bill that would have limited the number of patents attached to a biologic drug, rather than the small-molecule drugs discussed here, passed the Senate in the form of the Affordable Prescriptions for Patients Act of 2023, although it never reached a House vote.^29^ Nor has either body had the opportunity to consider a similar approach for small-molecule drugs.

In contrast, other nations and governing bodies have passed legislation to curb modification patenting: The EU prohibits patents on methods of treatment for therapy on the human body,^30^ and Israel prohibits formulation change patents and some chemical change patents.^31^ Section 3(d) of India's Patent Act bars patents on new forms of known substances unless they demonstrate meaningfully enhanced therapeutic efficacy, explicitly targeting the kind of modification claims this paper examines.^32^ At the international level, the United Nations Development Program has similarly recommended patent examination guidelines suggesting that many such modification claims should be scrutinized for lack of novelty or inventive step.^3^ We hope that by setting forth the frequency and protection-extending impact of modification patents per API, this paper may encourage greater policymaking attention.

## Supplementary Material

qxag123_Supplementary_Data
